# A Persistent Pulmonary Puzzle: Diagnosing Hodgkin Lymphoma in a Young Female With Chronic Respiratory Symptoms

**DOI:** 10.7759/cureus.65569

**Published:** 2024-07-28

**Authors:** Zaid Sawaftah, Ameer Awashra, Ali Bani Odah, Ahmed Sawafta, Abed Alawna, Jehad Khamaysa, Mohammed Abdalqader, Yazan Ghannam

**Affiliations:** 1 Department of Medicine, An-Najah National University, Nablus, PSE; 2 Faculty of Medicine and Health Sciences, An-Najah National University, Nablus, PSE; 3 Department of Radiology, Tubas Turkish Governmental Hospital, Tubas, PSE; 4 Department of Internal Medicine, Tubas Turkish Governmental Hospital, Tubas, PSE

**Keywords:** radiotherapy, persistent cough, reed-sternberg cells, necrotizing pneumonia, hodgkin’s lymphoma

## Abstract

Hodgkin lymphoma (HL), a lymphoid neoplasm characterized by the presence of Reed-Sternberg cells, often presents with painless lymphadenopathy and systemic symptoms. This case report details the diagnostic journey of a 27-year-old non-smoker female with chronic respiratory symptoms, including persistent cough, hemoptysis, and weight loss over two years. Despite multiple treatments for presumed infections and extensive diagnostic procedures, the correct diagnosis of HL was delayed due to atypical pulmonary manifestations, notably necrotizing pneumonia and multiple cavitary lung lesions. Ultimately, histopathology from a third bronchoscopy confirmed HL, highlighting the complexity of diagnosing HL with unusual presentations. Patients with cavitary lesions have a poor prognosis compared to others with typical pulmonary involvement, as cavitation in HL is likely caused by central ischemia necrosis due to the tumor's rapid growth. This case can be considered a primary pulmonary HL, a rare and hard-to-treat presentation since it does not respond well to radiotherapy. It emphasizes the challenge in diagnosing HL when it presents atypically, making it crucial to consider HL in differential diagnoses to avoid delayed diagnosis and improve patient outcomes.

## Introduction

Hodgkin lymphoma (HL), a lymphoid neoplasm known as Hodgkin’s disease, is defined by the presence of distinctive multinucleated and mononuclear Hodgkin/Reed-Stemberg (HRS) cells, which are considered pathognomonic. Studies in biology and medicine have separated this disease entity into two categories: nodular lymphocyte-predominant Hodgkin lymphoma (NLP-HL) and classical Hodgkin lymphoma (cHL). The clinical presentation and pathophysiology of these two disease entities differ from one another [1]. cHL is the more common category accounting for approximately 95% of all HL. With a high incidence among people in their 20s and a second, lower peak in older adults, it often affects young adults and is subdivided into four subtypes: nodular sclerosis Hodgkin lymphoma (NSHL), mixed cellularity Hodgkin lymphoma (MCHL), lymphocyte-depleted Hodgkin lymphoma (LDHL), and lymphocyte-rich Hodgkin lymphoma (LRHL). Patients in this category typically present with painless cervical lymphadenopathy and histological findings of cHL characterized by large abnormal multinucleated Reed-Sternberg cells [2]. However, NLP-HL accounts for about 5% of all HL cases and is more common in males and often presents in young to middle-aged adults. B symptoms are less common in NLP-HL with slow-growing lymphadenopathy. Histological findings of this category are characterized by lymphocyte-predominant (LP) cells, which are a variant of Reed-Sternberg cells [3]. Luckily, HL has an excellent overall prognosis with approximately an 80% cure rate. HL is mainly treated with chemotherapy depending on the histologic characteristics, the stage of the disease, and the presence or absence of prognostic factors [1,3]. Involvement of Hodgkin's disease in the lungs is not rare, and many previous cases have reported that Hodgkin's disease can mimic lung abscess or necrotizing pneumonia [2-4].

We report a case for a 27-year-old female with a significant past medical history of lung infection, who presented with recurrent productive cough, leading to the diagnosis of HL. Further investigations revealed necrotizing pneumonia and increased inflammatory markers. The patient was treated with different types of antibiotics and anti-fungi, showing minimal improvement, and was subsequently referred for an oncological consult for follow-up care and chemotherapy.

## Case presentation

A 27-year-old non-smoker female without a significant past medical history presented with a constellation of symptoms, including a persistent productive cough, hemoptysis, shortness of breath, fatigue, chest discomfort, anorexia, night sweats, and weight loss, all persisting over the past two years. On physical examination, the patient was found to be conscious, alert, and oriented, with stable vital signs, except for a slightly elevated temperature of 37.9°C. Notably, bilateral decreased air entry was detected upon auscultation of the lungs.

Over the previous two years, the patient had been treated with multiple courses of antibiotics without significant improvement, leading to further diagnostic investigations. Laboratory tests revealed elevated inflammatory markers (Table 1). A chest CT scan showed airspace consolidation in the left upper lobe with cystic spaces containing air and minimal fluid, alongside mild left pleural effusion with a maximum thickness of 2 cm. Multiple ground-glass opacities were scattered throughout both lung segments, and a few reactive mediastinal lymph nodes were noted, measuring up to 1.5 cm in size and characterized by a homogeneous enhancement pattern without necrosis. The possibility of tuberculosis (TB) was ruled out through negative sputum acid-fast bacilli staining, culture tests, and a TB polymerase chain reaction (PCR) test. Furthermore, sarcoidosis was considered unlikely due to the absence of non-caseating granulomas in transbronchial biopsy samples and a negative angiotensin-converting enzyme test.

**Table 1:** Laboratory values of inflammatory markers, laboratory results of vasculitis screen and serology, and pulmonary function test results. CRP: C-reactive protein; ESR: erythrocyte sedimentation rate; WBC: white blood cells count; C-ANCA: antineutrophil cytoplasmic antibodies; P-ANCA: perinuclear antineutrophil cytoplasmic antibodies; ANA: antinuclear antibodies; HIV: human immunodeficiency virus; ELISA: enzyme-linked immunosorbent assay; FVC: forced vital capacity; FEV1: forced expiratory volume in one second; TLC: total lung capacity; RV: residual volume.

Inflammatory marker	Result
CRP	175 mg/L
ESR	115 mm/hr
WBC	26000 cells/μL
Platelet count	520000 cells/μL
Ferritin	279 ng/mL
Vasculitis screen and serology	
C-ANCA	0.6 unit/mL
P-ANCA	0.7 unit/mL
ANA	1:2
Brucella test	Negative
HIV (ELISA)	Negative
Aspergillus fumigatus-specific immunoglobulin G antibodies	10 mg/L
Asparagus serum procalcitonin	0.07 ng/mL
Asparagus serum galactomannan index	0.2
Pulmonary function test	
FVC	2.06 L (52%)
FEV1	1.91 L (57%)
FEV1/FVC	(108%)
TLC	3.32L (63%)
RV	1.26L (109%)
RV/TLC	38%

In response to these findings, the patient underwent two bronchoscopic procedures for bronchoalveolar lavage and biopsy. These procedures revealed a chronic infection process, with cultures growing *Staphylococcus aureus* and *Klebsiella pneumoniae*, necessitating the continuation of antibiotic therapy. Despite this, the patient’s condition did not improve, and follow-up imaging indicated progressive radiological changes. These included complete cavitation of the left upper lobe, cavitating consolidation in the left lower lobe, and several cavitating nodules in the right lung. Other potential causes of multiple cavitating lung lesions, such as fungal infections (ruled out by negative galactomannan and *Aspergillus* antibody tests), TB (ruled out by negative sputum acid-fast bacilli staining, culture, and TB PCR), and malignancies (ruled out by repeated negative cytology and histopathology), were systematically excluded.

Given the lack of improvement and the progression of the disease, a vasculitis screen and serologies for rheumatic diseases were performed, all of which returned negative results (Table 1). The patient's pulmonary function tests (PFTs) revealed a significant decline in lung function (Table 1), prompting discussions about surgical intervention. The medical team proposed the removal of the left upper lobe cavity and a surgical lung biopsy to definitively rule out malignancy or vasculitis. The patient consented, and a wedge resection of the left lower lung was performed. Unfortunately, the pathology results were non-conclusive, showing only signs of chronic infection.

**Figure 1:**
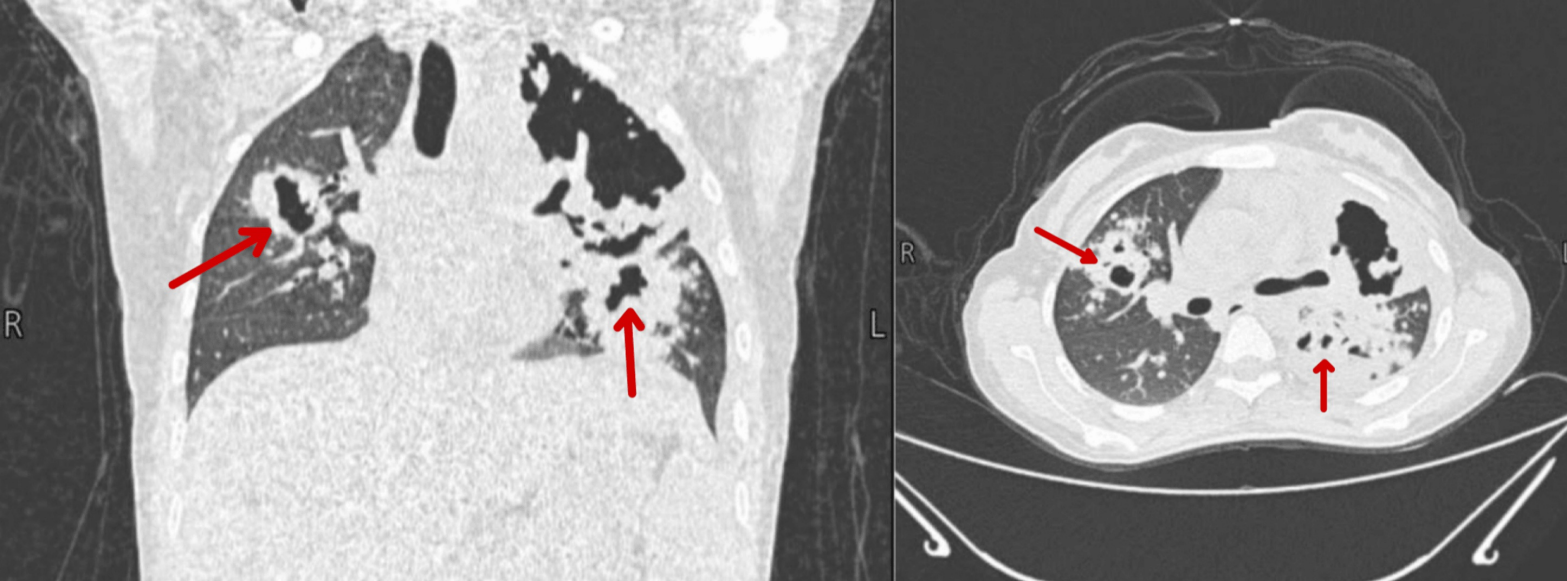
Chest CT scan revealed bilateral consolidation and a crazy paving pattern, accompanied by large cavities indicative of extensive parenchymal destruction, predominantly on the left side. Additionally, numerous small cavitating nodules were observed in all lobes, along with mild peripheral ground-glass opacity. There was also evidence of bronchial plugging at the lung bases.

Weeks later, the patient’s symptoms continued to deteriorate, characterized by worsening respiratory distress and further weight loss of approximately 4 kg over a period of two months. Consequently, a repeat chest CT scan demonstrated bilateral consolidation with a crazy paving pattern and large cavities, suggesting extensive parenchymal destruction, predominantly on the left side. Numerous small cavitating nodules were present across all lung lobes, accompanied by mild peripheral ground-glass opacity and bronchial plugging in the lung bases (Figure 1).

Subsequently, a third bronchoscopy was conducted, and histopathology finally revealed a diagnosis of HL explaining the chronic and recurrent nature of the patient’s symptoms and radiological findings. Therefore, the patient was subsequently referred to an oncology center for specialized care and treatment planning for her newly diagnosed HL.

Three months later, she reported some improvement in her symptoms, noting a decrease in the frequency of her night sweats and a slight increase in appetite. However, she continued to experience significant fatigue and intermittent shortness of breath. Her physical examination revealed stable vital signs, though she appeared more frail and had lost an additional 2 kg since her last visit. Auscultation of her lungs indicated persistent but stable bilateral decreased air entry, without new findings. Laboratory results showed a moderate improvement in her inflammatory markers, suggesting a partial response to chemotherapy. A recent PET-CT scan revealed a reduction in the size of the mediastinal lymph nodes and a decrease in metabolic activity within the nodules, indicating a positive response to the treatment. However, the scan also showed persistent cavitating nodules and ground-glass opacities in the lungs, with some new areas of consolidation, particularly in the lower lobes. Additionally, the team discussed the potential need for surgical intervention in the future if the cavitating nodules do not respond to medical management.

## Discussion

HL is a lymphoid neoplasm that affects approximately 9000 patients annually with a classic presentation of painless supradiaphragmatic lymphadenopathy with unique histologic features like the presence of Reed-Sternberg cells, which are considered pathognomonic. HL can be divided into two main types: NLP-HL and cHL. The latter can be subdivided into NSHL, MCHL, LDHL, and LRHL. Most patients (50%) present with painless rubbery enlargement of the superficial lymph nodes of the neck and supraclavicular area and systemic B-symptoms (fever, night sweats, and weight loss). However, it has a wide range of presentations [5,6].

Of the patients with HL, 9% have radiological evidence of parenchymal lung involvement at the time of diagnosis. Literature has described three radiological patterns: nodular, bronchovascular-lymphangitic, and pneumonic-alveolar [6,7]. Moreover, about 38% of all patients have pulmonary and pleural involvement throughout their disease course [8]. Lung involvement is almost always present with lymphadenopathy, thus a patient with a seemingly primary lung tumor or infection usually does not have HL in their differential, making it hard to diagnose early [6,9]. As in our case, the patient underwent many bronchoscopies with varying positive cultures, and took the medical staff about two years to get the proper diagnosis.

Previously, radiation therapy was used to treat HL. This technique was the only available option for HL patients [10]. Nowadays, the mainstay of therapy is the doxorubicin, bleomycin, vinblastine, and dacarbazine regimen, which has been effective for 30 years [11]. In the case of a localized bulky mediastinal tumor that occupies more than one-third of the mediastinal diameter, combined radiotherapy and chemotherapy are recommended if it is superior to either modality alone [12]. Currently, the prognosis of HL is one of the most curable malignancies, with a five-year relative survival rate that ranges between 89.8% and 96.4% among different age groups [13].

At the Ann Arbor Conference in 1971, scientists and physicians established the HL staging system, which was then modified by the Cotswolds Meeting in 1988. According to the four phases of the disease, the staging approach takes into account both the number of sites of involvement and whether the disease is present above or below the diaphragm. A thorough history that documents the occurrence and duration of any potential systemic symptoms, a precise physical examination, and comprehensive blood and biochemical tests are all part of the comprehensive staging work-up for HL [14].

Patients with cavitary lesions have poor prognosis compared to other patients with typical pulmonary involvement. In our case, the patient's lung involvement manifested as multiple cavitary lesions. It is a rare and hard-to-treat presentation since it does not respond well to radiotherapy. Previous investigations indicate that cavitation in HL is most likely caused by central ischemia necrosis [15]. This is probably because of the tumor's rapid growth and tendency to appear in big nodules and masses [16].

This case can be considered a primary pulmonary HL [16]. Many primary pulmonary HL cases have been described in the literature [15,17-20]. Nevertheless, our case is unique in that it is presented with multiple cavitary lesions in both lungs, making it extremely difficult to diagnose or to include HL in differential diagnoses.

## Conclusions

HL is a type of lymphoma that originates from B lymphocytes. This condition may produce a variety of signs and symptoms, among which multiple cavities in both lungs are considered to be a difficult presentation. In this particular case, the patient’s diagnostic delay involved multiple bronchoscopies and interventions for presumed endobronchial infections before reaching the proper diagnosis of primary pulmonary HL. This showcases the need to take into consideration HL in atypical manifestations to avoid delayed diagnosis, thus increasing the patient’s prognosis.
